# Nocturnal Lepidoptera Larvae in Mega‐Project: Habitat and Environmental Factors Over Construction Effects

**DOI:** 10.1002/ece3.74296

**Published:** 2026-09-29

**Authors:** D. Trombin, P. Mazzei, I. Piccini, L. Dessì, P. Grieco, E. Matechou, S. Bonelli

**Affiliations:** ^1^ Department of Life Sciences and Systems Biology University of Turin Turin Italy; ^2^ Associazione Lepidotterologica Italiana (ALI) Turin Italy; ^3^ Department of Zoology Poznań University of Life Sciences Poznań Poland; ^4^ Department for Sustainable Development and Ecological Transition University of Piemonte Orientale Vercelli Italy; ^5^ Tunnel Euralpin Lyon Turin (TELT) Torino Italy; ^6^ School of Mathematical Sciences Queen Mary University of London London UK

**Keywords:** ACI design, biodiversity conservation, construction site impacts, light pollution, moth larvae

## Abstract

Mega‐projects are large‐scale developments that profoundly transform landscapes while delivering infrastructure and socio‐economic benefits. However, they are also major drivers of biodiversity loss through multiple direct and indirect impacts. Among these, artificial light at night (ALAN) is increasingly recognised as a threat to biodiversity, including nocturnal Lepidoptera, which play crucial ecological roles. While ALAN effects on adult moths are well documented, its influence on preimaginal stages, critical for fitness and population persistence, remains poorly understood. This study examined construction‐related factors, including ALAN effects, on Lepidoptera larvae in natural environments within mega‐projects context using an After‐Control‐Impact (ACI) design. Focusing on larvae is essential, as this longest and least mobile life stage is particularly vulnerable to local environmental alterations such as artificial lighting, and environmental complexity can either buffer or amplify ALAN effects. However, our findings indicate that larval presence, species richness and biomass were not consistently associated with ALAN intensity. Instead, other environmental factors, including habitat characteristics, plant species, exposure and vegetation management, emerged as more important determinants of larval assemblages. In two sites, at least one control area showed higher larval abundance than the impacted site, but this pattern appears linked more to local environmental conditions than to lighting. Species richness showed no consistent pattern, and biomass analyses were limited by sample size and developmental variability. Overall, the results highlight that mega‐project impacts on nocturnal Lepidoptera larvae are complex and context‐dependent. ALAN was not retained as a significant predictor relative to other environmental factors, though this may reflect limited statistical power rather than a true absence of effect. These findings emphasise the need for standardised approaches to mitigate multiple construction‐related impacts on nocturnal Lepidoptera.

## Graphical abstract

This study assessed the effects of construction‐related factors, including ALAN, on nocturnal Lepidoptera larvae in mega‐project areas. The results suggest that local environmental conditions play a greater role than ALAN intensity in shaping larval assemblages.

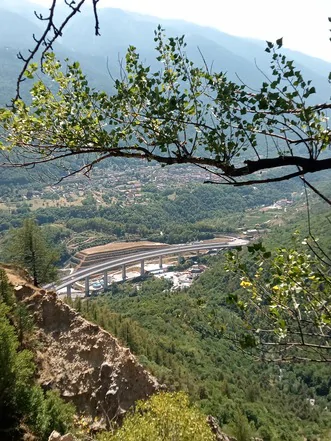

## 1 Introduction

Mega‐projects are extensive, long‐term initiatives (Flyvbjerg 2014) described as ventures that deliberately, deeply and visibly transform landscapes (Gellert and Lynch 2003). Their size continues to expand, following a persistent historical trajectory with no sign of decline (Flyvbjerg 2014). Characterised by prolonged planning and construction phases, these projects involve a broad spectrum of public and private stakeholders influencing the lives of millions of people (Thounaojam and Laishram 2022). They represent the preferred means for delivering goods and services across numerous sectors, including infrastructure, water and energy systems, industrial processing and mining (Flyvbjerg 2014). Such initiatives typically require investments of at least USD 1 billion or more, and they are capable of producing rapid and profound environmental changes (Flyvbjerg 2014). Their expansion often generated direct and indirect environmental impacts (Laurance et al. 2015), and future infrastructure decisions may further threaten biodiversity and sustainability (Van Rees et al. 2023). Although frequently portrayed as environmentally friendly, they remain a major driver of biodiversity decline (Benítez‐López et al. 2010; Laurance et al. 2015), due to their intensive use of natural resources and the potential for large‐scale ecological damage (Brock 2023). Evidence shows that such developments can disrupt landscape connectivity and generate wide‐ranging impacts on biodiversity (Hou et al. 2024). Their construction frequently leads to habitat loss, as vast natural areas are converted into engineered environments, resulting in ecosystem destruction and increased fragmentation (Güneralp et al. 2021; Sloan et al. 2019). Mega‐projects also introduce multiple forms of pollution with serious ecological consequences. Emissions from heavy machinery and vehicles release greenhouse gases and particulate, negatively affecting biodiversity and human health (Cheriyan and Choi 2020; Piccini et al. 2023). Large‐scale construction may alter river systems through chemical contamination and damage to aquatic communities (Wu et al. 2019), while soil degradation from fuel spills and waste disposal can reduce fertility and contaminates groundwater (Sandil and Kumar 2022). Beyond these factors, noise and light associated with construction activities may also disturb nocturnal species. In particular, Artificial Light at Night (ALAN) disrupts circadian rhythms and reproductive behaviour, adding stress to already vulnerable wildlife populations (Gili et al. 2024; Ojija et al. 2025). Alterations in ambient light levels represent one of the fastest‐growing forms of environmental change (Cinzano et al. 2001). Artificial lighting is increasing globally by about 6% per year (Hölker et al. 2010), intensifying light pollution worldwide, including across Europe (Bennie et al. 2014; Guetté et al. 2018). Light pollution is primarily associated with urbanisation, with dense population and urban agglomerations (Cinzano et al. 2001; Falchi et al. 2019; Kumar and Shaw 2020). Infrastructure construction and other mega‐projects contribute to the rise in artificial illumination, both during construction and operational phases. ALAN in construction sites serves multiple purposes, including worker safety, equipment operation and night‐time activities. These projects often introduce high‐intensity lighting into remote, biodiverse areas previously characterised by low or absent light pollution. Unlike urban centres, where biodiversity is already reduced, such sites frequently support high levels of naturalness and species richness. The biological effects of ALAN are considerable (Gaston et al. 2014; Perkin et al. 2011) affecting several taxa—including mammals, birds, reptiles, amphibians, fish, invertebrates and plants—across terrestrial and aquatic ecosystems (Gaston et al. 2014). ALAN alters physiology, behaviour, reproduction and movements (Dominoni et al. 2013; Stone et al. 2009), and influences species abundance, distribution, community structure and ecosystem functioning (Davies et al. 2012; Meyer and Sullivan 2013). Most light‐affected organisms are nocturnal, and numerous negative effects have been described among insects (Owens et al. 2020), particularly given that around 64% of invertebrate are nocturnal and many are declining globally (Hölker et al. 2010). Artificial light plays a key role in the current declines of nocturnal Lepidoptera (Bennie et al. 2018; Van Grunsven et al. 2020), which respond rapidly to environmental changes in both community composition and phenology (Maes and Dyck 2001; Thomas 2005). ALAN can impact various life stage of nocturnal Lepidoptera (Boyes et al. 2021a): oviposition (Macgregor et al. 2017), larval growth and feeding (Boyes et al. 2021b; Loewy et al. 2013), pupation and its duration (Van Geffen et al. 2015), adult activity (Macgregor et al. 2017) and reproduction (Péter et al. 2020). Furthermore, artificial light can impair dispersal and contribute to habitat fragmentation (Degen et al. 2016).

This study aimed to investigate the larval stage of nocturnal Lepidoptera to explore the potential effects of mega‐project construction sites, with particular focus on ALAN effects on larval feeding rates. It represents a field‐based attempt to examine ALAN impacts on Lepidoptera larvae in the natural environment within the context of international mega‐projects. Nocturnal Lepidoptera were selected as an indicator group for wider invertebrate biodiversity, given their ecological relevance and sensitivity to environmental change. Our objective was to identify key environmental factors during large‐scale infrastructure construction and evaluate their effects on nocturnal Lepidoptera communities, with particular attention to the larval phase. This life cycle phase was selected as it represents the longest life stage, depends on vegetation, and involves limited mobility, making larvae highly sensitive to local conditions (Zalucki et al. 2002). Moreover, adult fitness largely depends on larval performance and pupal weight, which determine survival and reproductive success (Boggs and Freeman 2005; Zalucki et al. 2002). To this end, we compared control areas and construction sites, focusing on the larval stage, which has received far less attention than the adult phase.

## 2 Materials and Methods

We conducted the study in two Alpine valleys (Figure 1): the Susa Valley (Western Italian Alps, Piedmont) and the Haute Maurienne‐Vanoise Valley (French Alps, Auvergne‐Rhône‐Alpes). Both areas are involved in the construction of the Turin‐Lyon high‐speed railway managed by TELT (Tunnel Euralpin Lyon Turin; Figures S1–S3, Supporting Information). The sites include the main tunnel excavation and other works, all illuminated by powerful night‐time lighting to enable continuous operations and ensure worker safety. The valleys differ in altitude and habitat (Figure S4–S5). The Susa Valley (638–1088 m a.s.l.), crossed by the Dora Riparia River, hosts natural and semi‐natural forests dominated by abandoned chestnut orchards, oaks and cherry trees. In contrast, the Haute Maurienne‐Vanoise Valley (1072–1334 m a.s.l.), crossed by the Arc River, is mainly composed of coniferous forests interspersed with xeric south‐facing pastures under low annual rainfall. For each construction site, we selected unlit control areas (Table 1, Figure 1) based on comparable habitat features, including exposure, altitude, land use, geomorphology and vegetation. Two construction sites were paired with three control areas, while one site (Area 2) had only one control area due to high urbanisation and artificial lighting. The three construction sites were characterised by different elevation, exposure, habitat type and artificial light intensity. Area 1 was located in an altitudinal range of 1110–1334 m a.s.l. in a dry ecotone habitat. Instead, Area 2 was placed between 1072 and 1099 m a.s.l. in the riparian habitat. Area 3 was characterised by an ecotone habitat type and located between 638 and 1088 m a.s.l. In contrast, within each group, habitat type, elevation and dominant vegetation composition did not differ substantially between the construction site and its associated control areas (Table S1). Light intensity was measured in all sites using a HoldPeak HP‐881E digital lux meter (resolution = 0.01 lx) to verify ALAN levels. The maximum value of artificial light was registered in Area 1 (35.2 lx), followed by Area 3 (20.6 lx) and Area 2 (13.0 lx). The average ALAN recorded at each construction site depended on the positions of the sampled trees. We noticed that different sampling points had different artificial light values (Table S2). The highest average was recorded in Area 1 (5.33 lx ± SE 0.442), followed by Area 2 (3.30 lx ± SE 0.453) and finally Area 1 (2.04 lx ± SE 0.591). These continuous light measurements were used to test for a dose‐dependent effect of ALAN intensity independently of site classification (control or construction site).

**FIGURE 1:**
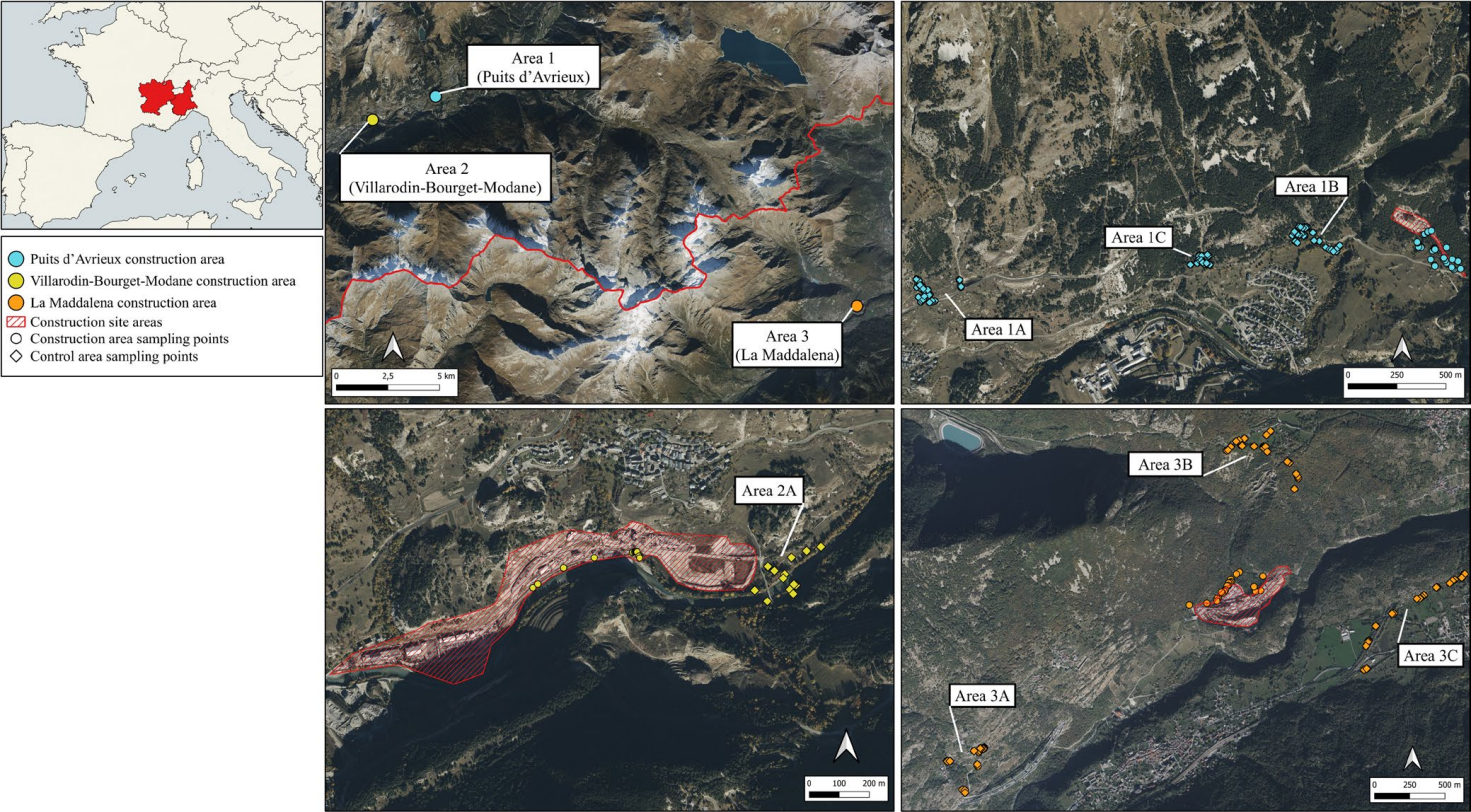
Map of the three study areas and the corresponding sampling points, divided into control areas (C) and construction site areas (T). Top left: Territorial overview of the construction sites under study. Top right: Avrieux construction site; bottom left: Villarodin‐Bourget‐Modane construction site; bottom right: La Maddalena construction site.

**TABLE 1:** Mega‐project construction sites and the control areas associated with the abbreviation for each sampling area. The same number of sampling points was investigated in construction site and each associated control area.

Country	Mega‐project construction site	Number of sampling points	Control areas
*France*	Area 1 (Puits d'Avrieux)	*N* = 28	Area 1A (Le Bourget) Area 1B (Cascade St. Benȏit) Area 1C (Soleil)
*France*	Area 2 (Villarodin‐Bourget‐Modane)	*N* = 17	Area 2A (Villarodin‐Bourget‐Modane_1)
*Italy*	Area 3 (La Maddalena)	*N* = 23	Area 3A (La Ramats) Area 3B (Pian de Ruine) Area 3C (Gravere)

The applied experimental approach allowed us to compare larval presence/absence at the sampling‐unit level considering all species detected, species richness and total biomass sampled in the lit construction site with the other three areas characterised by low or completely absent lighting levels. The field experiment was based on a modified version of Before/After‐Control Impact approach (*BACI*), which foresees sampling in a disturbed area and in a set of control areas, before, during and after the impact. In our case, the impact of construction operations began several years before our study started, although the timing differed among areas. Construction activities started in 2007 in Villarodin‐Bourget‐Modane, in 2013 in La Maddalena site, and in 2017 in Puits d'Avrieux. Consequently, we used an *After‐Control/Impact* approach, described and compared with other approaches by Christie et al. (2019), that allowed us to investigate the effect of ALAN using a set of control areas instead of the classical method applied in impact evaluations, which does not include any control area.

Different numbers of sampling points were selected for each area because of the different habitat and vegetation type characteristics (maps of sampling points per each area are available in Supporting Information, Figures S6–S8). Each sampling point corresponded to a single plant selected for larval sampling. We selected 28 sampling points in Area 1 (Puits d'Avrieux), 17 sampling points in Area 2 (Villarodin‐Bourget‐Modane) and 23 sampling points in Area 3 (La Maddalena); the same number of points, as their corresponding treatment sites, were sampled in each control area (Table 1). Different plant species were included in the sampling (Table S3), and their selection was based on knowledge of larval stage preference in terms of trophic resources. All selected plants were identified by an expert; furthermore, each plant was measured (height, diameter and circumference) to choose and standardise plant dimension.

Larval sampling was conducted across 187 sampling points using vegetation beating and net sweeping following the entomological umbrella net principle (Basset et al. 1996) on the target plant species. Each study area was monthly surveyed four times from June to October 2022. The collected larvae were weighed using an analytical balance (Balance CT‐33 Digital Scale, precision: 0.001 g) and then released on the same plant. The individuals were weighed to assess larval fitness, using both their weight and developmental stage as indicators of foraging rate. All larvae were photographed in the field, and species' identification was subsequently performed by an expert (Paolo Mazzei), coauthor of this work. Identification and developmental assessment were based on distinctive morphological features and on ecological knowledge of the species occurring in the study area, including their distribution and phenology. The morphological traits examined for species identification included: general morphological peculiarities, distribution and density of setae, dorsal, lateral and ventral colour patterns, shape and pigmentation of spiracles, shape and colour of the head capsule. Due to the challenges encountered during the species' identification phase, we assigned morpho‐species to specimens that were certainly not previously identified but for which a definitive identification could not be achieved.

### 2.1 Statistical Analysis

The analyses were run using R (v. 2024.09.0.375, RStudio: Integrated Development Environment for R Posit Software). The larval presence/absence, species richness and the total larval biomass were considered as response variables in separate statistical models. All continuous variables were scaled and centred to make effect sizes directly comparable. Moreover, we tested collinearity between variables using the threshold of *r* > 0.6 (Zuur et al. 2009). To assess multicollinearity among predictor variables, we used the *checkcollinearity function* from the performance package (Lüdecke et al. 2021). This function calculates variance inflation factors, which were examined to ensure that collinearity did not affect model performance. Several environmental covariates were included in each model (Table S4). For the analysis of species richness and biomass, a reduced set of variables was considered compared to the full set of covariates used for the analysis of larval occurrences (Table S5–S6). Furthermore, we considered the larvae developmental stage which can significantly influence the weight of the specimen sampled.

The distribution of larvae count data was heavily zero‐inflated, with very few counts greater than one. For this reason, we converted the data to binary form, indicating species presence (1) or absence (0) for each surveyed plant. A table of frequencies is provided (Table S7) to support this decision. The binomial distribution was used to analyse the presence/absence data. Species richness was calculated by counting the number of species per point and was assigned a zero‐inflated negative‐binomial distribution. We calculated the larval biomass per area summing the weight of all larvae sampled on each plant species per area and removing an outlier belonging to *Saturnia pyri* (Denis and Schiffermüller, 1775) that is a very large species found on one occasion during the sampling. The biomass data were characterised by small decimal numbers, few observations (*N* = 40) and many zeros. For this reason, a zero‐inflated Gamma distribution was used to model the data.

The ‘glmmTMB’ package (Brooks et al. 2017) was used for all three analyses and the ‘DHARMa’ package (Hartig 2016) was used to create readily interpretable scaled residuals for fitted generalised linear mixed models. The forward selection of explanatory variables (Harrell 2015) was employed using the Akaike's Information Criterion (AIC) (Akaike 1973). Model predictions were obtained using the *predict* function in R with type set to “response” and visualised using ggplot2 (Wickham et al. 2016). The *emmeans* function was used to obtain the response for each level of the variables included in the model. We provided the fitted models and relative AIC values (Table S8–S11). Different random effects were considered in order to account for potential variations due to repeated sampling within the same areas, sampling points and plant species. For the presence/absence and species richness analysis, we tested different combinations of the three random effects (area, point, sp_veg), assessing their variance in the models, as this analysis was conducted at the sampling point level. In contrast, the biomass data were aggregated at the area level, for this reason “point” could not be used as a random effect. As a result, we included different random effects in the three analyses: “point” for presence/absence and species richness, and “area” for total biomass.

## 3 Results

### 3.1 Larvae Presence‐Absence Models

In total, we collected 248 individuals: 50 individuals from Area 3, 167 from Area 1 and related controls, and 31 from Area 2 and its controls. The highest number of larvae was sampled in July, corresponding to the seasonal peak for the taxon. Area 1B hosted the most individuals, followed by construction site Area 1 (Figure S9). *Betula pendula* Roth, 1788 hosted the largest number of larvae (*N* = 51), followed by *Berberis* spp. (Linnaeus, 1753) (*N* = 45); while other plant species hosted no more than 31 individuals.

The model selected by AIC at the end of the forward selection process for the presence/absence data (Table 2) revealed that the larval occurrence was influenced by area (Figure 2), mowing (Figure S10), plant species (Figure S11) and wind (Figure S12). ALAN was tested as a univariate predictor prior to forward selection (Table S12) and showed a negative but non‐significant effect on larval occurrence; it was not retained in the final model. Significant differences were detected when comparing sampling areas using the *emmeans* function. Construction site Area 1 showed higher larval occurrence than its control Area 1C (Est = 0.370, *p* = 0.0002), while no significant differences emerged between Area 1 and its other controls (Area 1A, Area 1B), nor between Area 2 and its control Area 2A, or Area 3 and its controls (Area 3A, Area 3B, Area 3C). Larval presence was significantly higher in recently mown areas (new; Est = 1.091, *p* = 0.001) than in unmown areas. Plant species strongly influenced larval presence: compared to *Rosa canina* (Linnaeus, 1753), higher occurrences were recorded on *Berberis* spp. (Est = 2.710, *p* < 0.001), *Betula pendula* Roth, 1788 (Est = 0.833, *p* = 0.021) and *Rubus* spp. (Est = 1.241, *p* = 0.031).

**TABLE 2:** Results of presence/absence GLMM. Model estimates ± SE and *p*‐values are given. *p*‐values less than our chosen significance level (5%) are indicated with bold and with asterisks.

Best model structure				
Mod5 < − glmmTMB (pa ~ area + mow + sp_veg + wind (1|point), family = “binomial”, data = data4)				
Covariate	Estimate	SE	*Z*	Pr (> |*z*|)
Intercept	−0.9486	0.6767	−1.402	0.161
**Area_1A**	**−1.08517571**	**0.4758**	**−2.281**	0.023*
Area_1B	0.4651	0.4890	−0.951	0.34152
**Area_1C**	**−2.5332**	**0.5598**	**−4.525**	< e‐06***
**Area_2**	**−1.51539**	**0.6685**	**−2.267**	0.023*
Area_2A	−0.7908	0.6168	−1.282	0.199
**Area_3**	**−2.8772**	**0.6514**	**−4.417**	< e‐05***
**Area_3A**	**−2.9253**	**0.6817**	**−4.291**	< e‐05***
**Area_3B**	**−1.4041**	**0.6005**	**−2.338**	0.019*
**Area_3C**	**−2.6087**	**0.6629**	**−3.936**	< e‐05***
**Mow_new**	**1.0910**	**0.3336**	**3.270**	0.001**
Mow_old	−0.4758	0.3352	−1.420	0.156
Sp_Alnus incana	1.0164	0.8365	1.215	0.224
Sp_Amel_ovalis	0.7601	0.5051	1.505	0.132
**Sp_Berberis_spp**.	**2.7103**	**0.5254**	**5.159**	< e‐07***
**Sp_Bet_pendula**	**0.8326**	**0.3600**	**2.313**	0.021*
Sp_Cra_monogyna	0.8039	0.5145	1.562	0.118
Sp_Fra_excelsior	0.6055	0.4304	1.407	0.159
Sp_Hip_rhamnoides	1.3658	0.9297	1.469	0.142
Sp_Populus_spp.	−0.2632	0.9546	−0.276	0.783
Sp_Quercus_spp.	0.2377	0.6498	0.366	0.715
**Sp_Rubus_spp**.	**1.2412**	**0.5752**	**2.158**	0.031*
**Sp_Salix_spp**.	**2.2047**	**1.1465**	**1.923**	0.054*
Wind_mild	0.3363	0.4978	0.675	0.499
Wind_moderate	−0.3775	0.5686	−0.664	0.507
Wind_strong	−0.6884	0.5969	−1.153	0.249
Wind_verystrong	−0.9093	0.6717	−1.354	0.176

*Note:* Asterisks indicate statistical significance: *p < 0.05, **p < 0.01, ***p < 0.001.

**FIGURE 2:**
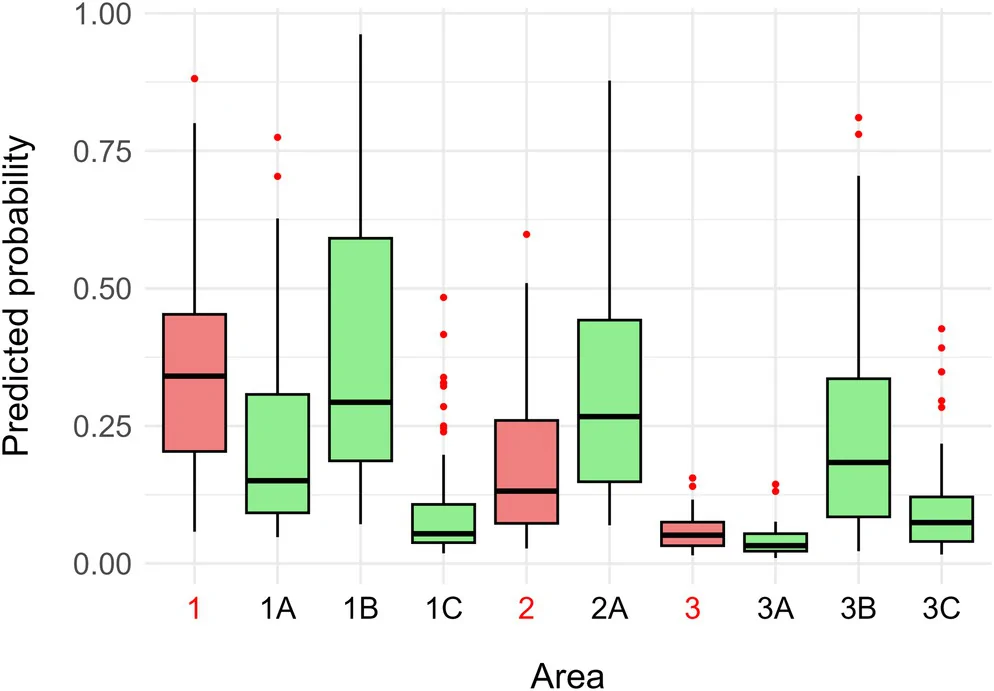
Predicted probability of moth larvae presence for each area; the construction site labels are in red character and associated control sites have green coloured boxplots.

### 3.2 Species Richness

We obtained 98 species, including 11 families and 42 genera (Table S13). The most represented family was Geometridae (*N* = 97), followed by Noctuidae (*N* = 31). The genus most frequent was *Diurnea* Haworth, 1811 (*N* = 27), and the most common species was *Diurnea fagella* (Denis and Schiffermüller, 1775) (*N* = 15). The highest species richness was detected in Area 1B (*N* = 26). In Area 2, species richness was considerably lower compared to the control area (Area 2A), whereas in Area 1 and 3 it was comparable to that of their respective control sites (Figure S13). The major number of species was found on *Betula pendula* (*N* = 29), followed by *Rosa canina* (*N* = 20), while all other species showed fewer than 20 species associated.

The best model (Table 3) suggests that species richness was significantly influenced by area (Figure 3), plant status (Figure S14) and sampling session (Figure S15). Using the *emmeans* function for the sampling area variable, Area 1 showed a higher species richness (Est = 0.26, *p* = 0.034) than the control Area 1C. The comparison between the construction site Area 1 and the construction site Area 3 indicated a higher species richness in Area 1 (Est = 0.250, *p* = 0.070). No other comparisons showed significant differences. Plant status significantly affected richness, with defoliated plants supporting fewer species (Est = −1.23, *p* = 0.042) than green and foliated plants. Additionally, species richness was higher during the third sampling session (Est = 0.59, *p* = 0.016) compared to the first. ALAN was tested as a univariate predictor prior to forward selection (Table S14) and showed a negative but non‐significant effect on species richness; for this reason, the covariate was not retained in the final model.

**TABLE 3:** Results of species richness GLMM. Model estimates ± SE and *p*‐values are given. *p*‐values less than our chosen significance level (5%) are indicated in bold and with asterisks.

Best model structure				
Mod23 < − glmmTMB (n_sp ~ area + mow + status_pl + repl + (1|point), family = “nbinom1”, ziformula ~1, data = rich_point)				
Covariate	Estimate	SE	*Z*	Pr (> |*z*|)
Intercept	−1.2488	0.2565	−4.868	< e‐06
**Area_1A**	**−0.5951**	**0.3100**	**−1.919**	0.054*
Area_1B	−0.1809	0.3079	−0.588	0.557
Area_1C	−1.6402	0.4179	−3.935	< e‐05
**Area_2**	**−0.8533**	**0.4114**	**−2.074**	0.038**
Area_2A	−0.1776	0.3725	−0.477	0.633
**Area_3**	**−1.7346**	**0.4637**	**−3.741**	0.001**
**Area_3A**	**−2.1991**	**0.5563**	**−3.953**	< e‐05***
Area_3B	−0.5559	0.3448	−1.612	0.106
**Area_3C**	**−1.4054**	**0.4161**	**−3.378**	0.001**
Mow_new	0.4341	0.3190	1.361	0.174
Mow_old	−0.3855	0.2588	−1.489	0.136
**Status_defoliated**	**−1.2318**	**0.6073**	**−2.028**	0.043*
Status_dry	−0.3703	0.3077	−1.203	0.229
Repl_2	0.3363	0.2737	1.229	0.219
**Repl_3**	**0.5928**	**0.2482**	**2.388**	0.017*
Repl_4	0.1594	0.2563	0.622	0.534

*Note:* Asterisks indicate statistical significance: *p < 0.05, **p < 0.01, ***p < 0.001.

**FIGURE 3:**
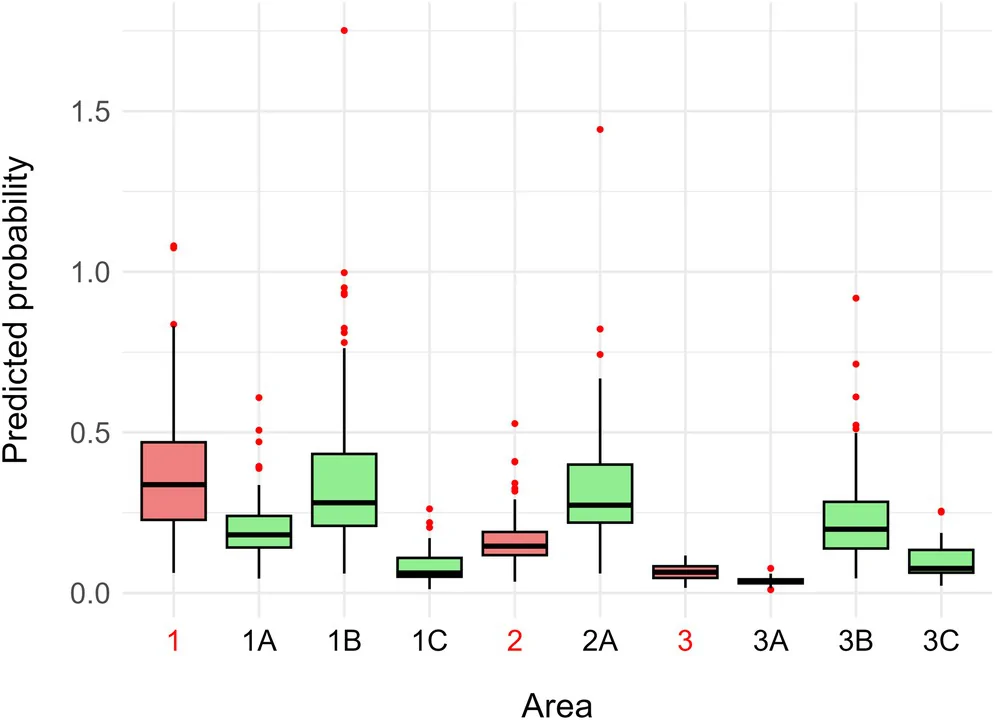
Predicted species richness along sampling areas. Construction sites are in red character and associated control sites have green coloured boxplots.

### 3.3 Larval Biomass

Larval weight exhibited considerable heterogeneity between sampled areas. Most larvae were small, likely due to the species encountered and the predominance of early developmental stages. Considering all sampling sessions, the maximum larval weight (1.050 g) was recorded in construction site Area 1; instead, in its control areas, the maximum was 0.760 g. Comparing the Area 2 site and its controls, the maximum larval weight was found in the control area (0.540 g). A similar trend emerged in the Italian sites, with the maximum weight recorded in Area 3C (0.715 g). Analysing the mean larval weight, we found low values across all sampling areas ranging between 0.0184 g and 0.1571 g, highlighting the small dimension of the sampled larvae.

Larval biomass was higher in Area 1 compared to its controls and the other construction sites, while it was similar between Area 2 and its control. In contrast, control Area 3B and 3C showed higher biomass than Area 2 (Figure 4). The lowest biomass was recorded in the construction site Area 3. Exploratory analyses of larval biomass distribution among plant species (Figure S16) showed that the highest biomass occurred on *Populus* spp., followed by *Salix* spp., *B. pendula* and *C. monogyna* . The plant species with lowest biomass were *Amelanchier ovalis* Medik, 1793 and *Berberis* spp.

**FIGURE 4:**
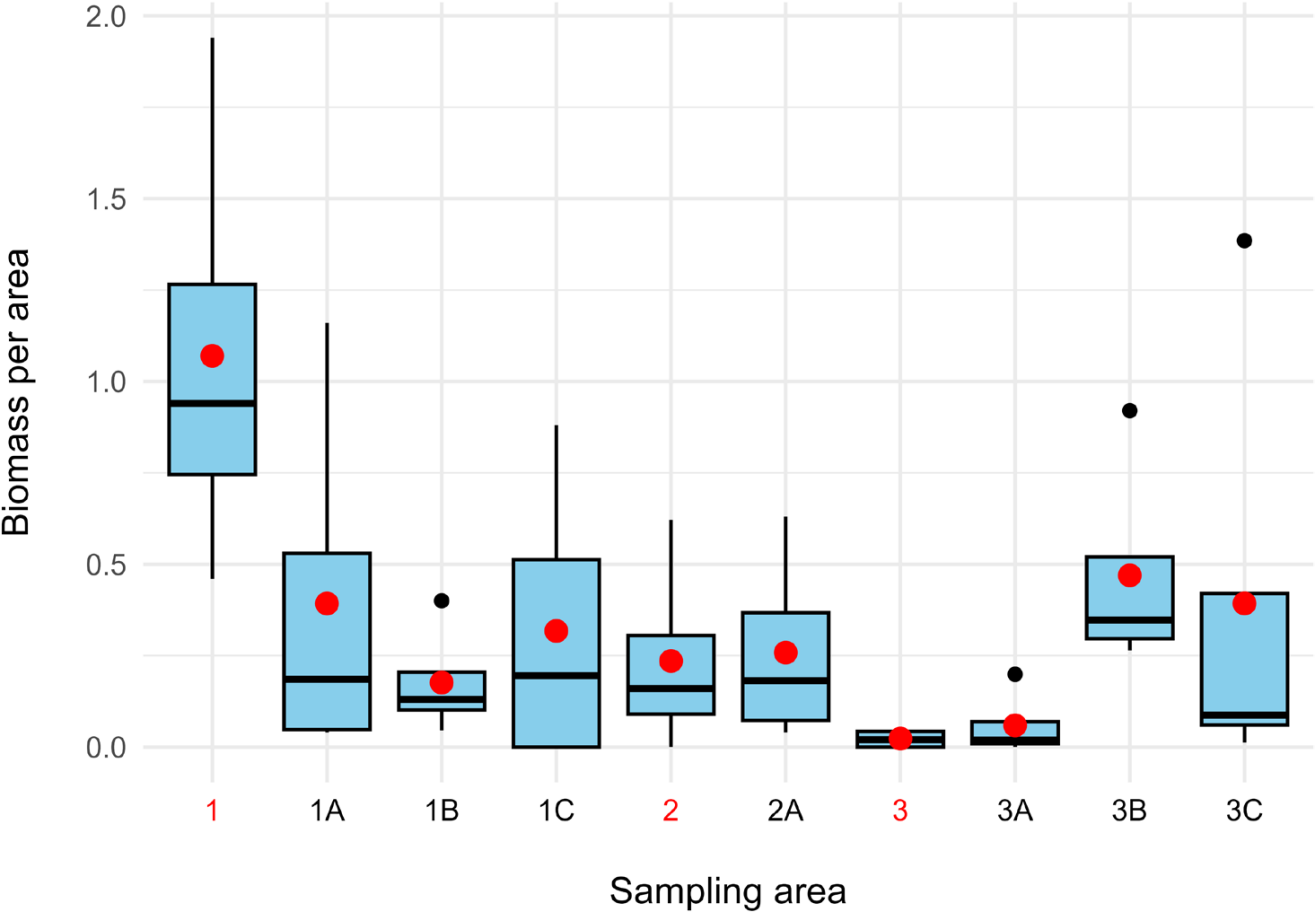
Distribution of biomass in different study areas; the red labels indicate construction sites, instead the red dots represent the mean value of biomass per area.

The forward selection process identified the null model as the best‐fit, indicating that none of the tested covariates significantly explained biomass variation. Across all tested models, the addition of predictors did not improve model performance according to AIC values. Consequently, no variables were retained in the final model, and biomass variation remained unexplained by the tested covariates.

## 4 Discussion

So far, the only available knowledge on nocturnal Lepidoptera in the French sampling areas is provided by specialised guides (Robineau 2011; Nel 2007) and the webpage lepinet.fr which reports the moth species occurring in these regions (Hautes‐Alpes). For the Italian area, the main references are the work by Piccini et al. (2023), which focused on the adult stage of this group. The species observed in the present research correspond to those reported in the cited studies, and no previously unrecorded species were identified.

Although the original aim of this study was to investigate the effects of ALAN on Lepidoptera larvae, our results indicate that other environmental factors—including habitat type, vegetation composition and local management practices—were retained as significant predictors in the final models, while ALAN was not. This should not be interpreted as definitive evidence that ALAN has no effect on larval stage: sparse count data likely limited the statistical power to detect an effect of artificial light. Our field research identified the highest occurrences at the French sites, particularly in Area 1 and 1B. In terms of statistical comparisons, the only significant contrast between a construction site and its own control was found between Area 1 and its control Area 1C, with higher larval occurrence in the construction site itself; no other significant differences emerged between any construction site and its associated controls.

Habitat structure likely influenced larval occurrences, particularly in Area 1 and its controls, which receive higher solar radiation and have a dry, warm xerothermic climate. Such conditions may favour adult moths' activity, which correlates with temperatures (Hellmann and Bertaccini 2004; Holyoak et al. 1997) and elevation (Neff et al. 2024). Our models further indicated a positive association between larval occurrence and recent mowing, contrasting with studies reporting reduced arthropod abundance in mown areas (Berger et al. 2024). However, the extent of this effect varies depending on the type of organism considered and its behavioural traits (Van Klink et al. 2019). We assumed that the result may reflect the role of vegetated margins adjacent to mown patches in supporting nocturnal Lepidoptera by mitigating management impacts, demonstrated by Merckx et al. (2012). Larvae were significantly more frequent on *Berberis* spp., *Rubus* spp. and *Betula pendula* species, consistent with previous findings (Coruh and Ozbek 2002; Kirichenko et al. 2017; Twidle et al. 2022).

Analyses of species richness mirror patterns in larval occurrence: the only significant area comparison in both models involved Area 1 and its control Area 1C, with higher values recorded in the construction site. Otherwise, no significant differences emerged between construction sites and their respective controls. Area 3 exhibited lower richness than Area 1, indicating that Area 1 supports higher larval occurrence and species richness. Species richness was higher on green plants than on defoliated ones, reflecting that many plants lost leaves prematurely during a prolonged drought, potentially forcing larvae to seek alternative food. Furthermore, the third sampling session in August showed higher richness than the first in June, likely due to the peak period of nocturnal moth activity and larger specimen size facilitating identification.

Regarding the analysis of biomass per sampling area, forward selection identified the null model as the best fit, likely due to limited statistical power. Data sparsity and low variability may have restricted the detection of meaningful predictors, and the environmental factors considered may not have exerted strong or consistent effects. Measured larval weights were extremely low, as many specimens were in the early larval stages, making them hard to detect and sometimes impossible to weigh. Although observation error was not explicitly modelled, it was likely consistent across surveys, sampling points and temporal replicates.

Despite intensive field sampling and a robust approach derived from the Beyond‐BACI design (Benedetti‐Cecchi 2001; De Palma et al. 2018; Underwood 1994), we encountered significant challenges. The approach was adapted to sites where impacts were already underway, and multiple controls were used to account for spatial and temporal variability (De Palma et al. 2018; Underwood 1992). Nevertheless, no clear trend or marked differences between controls and construction sites were observed in terms of presence/absence and species richness. These findings may reflect several factors. Firstly, the sample size of collected larvae was relatively small compared to similar studies (see Boyes et al. 2021a, 2021b). Larvae belonged to multiple species and developmental stages, and many could not be identified due to their small size and early instars, constraining biomass assessment—A key indicator of feeding rates and potential ALAN effects. Additionally, the intensity and distribution of ALAN varied across sites, with tree canopies partly obstructing light and producing uneven illumination. Similarly, the degree of ALAN exposure varied with plant location, with some plants fully illuminated and others partially shaded, potentially affecting larval occurrence and species richness. A further limitation concerns the characterisation of ALAN itself: light intensity was measured as lux values only, without capturing spectral composition, wavelength, colour temperature, or photoperiod, all of which are known to strongly influence insect physiology and behaviour (Trombin et al. 2026) independently of intensity alone. Future studies should incorporate spectral measurements to better characterise the specific components of ALAN driving ecological responses.

While tree beating is generally effective, accessing the full canopy is often difficult, leading to potential under‐sampling. Furthermore, preliminary trials showed that larvae became less likely to fall after the first impact during the beating procedure, reinforcing their grip to avoid dislodgement. This behaviour, exacerbated under moderate to strong winds, may have contributed to underestimating larval presence and abundance during the sampling.

Preliminary trials indicated that time of day did not influence larval detectability, suggesting that activity patterns had little impact on sampling efficiency. Nonetheless, behavioural and ecological factors may influence larval presence. Some moth species adjust feeding times to reduce predation risk (Abrams et al. 1996; Sibly et al. 1985), while foraging strategy can also vary with food availability, habitat and develop metal stage (Clark 1994; Gilliam and Fraser 1987). Predation risk is generally higher during the day when predators are active (Kronfeld‐Schor and Dayan 2003), particularly for larger larvae, whereas smaller larvae are more heavily preyed upon by both diurnal and nocturnal arthropods (Kristensen 1994; Zalucki et al. 2002). Since our study observed mostly, it is important to note that their mortality risk likely varies little over the 24‐h period (Zalucki et al. 2002). Berger and Gotthard (2008) showed that daytime feeding decreases with larval size in some diurnal Lepidoptera, whereas night time feeding remains high across all sizes. Temperature also influences larval growth, which is faster at higher temperatures (Angilletta et al. 2003), although extreme daytime heat could force larvae to seek refuge in lower parts of host plants (Reavey 1993; Slansky Jr. 1993). In summer 2022, severe drought and high temperature caused premature leaf yellowing and defoliation in shrubs and trees (e.g., *Rosa canina* , *Rubus* spp., *Quercus* spp.) in several areas (Area 1, 1C, 3A, 3C). Larvae are particularly sensitive to heat and drought, which reduce plant quality and can negatively affect species diversity, especially during vulnerable stages such as the larval phase as reported by Uhl et al. (2022).

## 5 Conclusion

Nocturnal Lepidoptera play a crucial ecological role as herbivores, pollinators and prey for birds and bats (Wickramasinghe et al. 2004), yet global declines have been widely reported (Fox 2013; Franzén and Johannesson 2007; Groenendijk and Ellis 2011), potentially causing cascading effects across trophic levels (Wickramasinghe et al. 2003). ALAN is recognised as a contributing factor, fragmenting habitats and impairing adult dispersal (Degen et al. 2016), which also affects preimaginal stages. Changes in adult mobility and distribution inevitably influence preimaginal stages which represent the longest phases of the life cycle; however, these remain poorly studied (Bennie et al. 2018; Van Grunsven et al. 2020).

Our results show that a significant difference in larval occurrence emerged only between one construction site and its control, with higher occurrence recorded in the construction site itself, indicating that the relationship between construction activity and larval presence is not straightforward. Species richness follows the same pattern, and biomass analyses were limited by small sample sizes and variability. Covariate analysis indicated that multiple factors, including vegetation structure, microclimatic conditions and local habitat management, shape larval presence and species richness, highlighting the complexity of assessing ALAN effects in the field. Habitat complexity may buffer or amplify light impacts, contributing to the contrasting results reported in previous studies (Boyes et al. 2021b; Grenis and Murphy 2019; Van Geffen et al. 2015; Péter et al. 2020). This study emphasises the importance of investigating high‐value natural areas typically free from light pollution, which serve as essential reference sites for understanding ALAN effects on nocturnal Lepidoptera. Most studies focused on urban environments with intense light pollution (Bates et al. 2014; Boyes et al. 2021b; Merckx and Van Dyck 2019; Nagy Pintérné and Pödör 2017) or suburban contexts (Grenis et al. 2015; Péter et al. 2020). Expanding research to semi‐natural areas is crucial to capture the full variability of ALAN effects across different habitats. Effects may be particularly pronounced for taxa or life stages with limited mobility. Other synergistic stressor, including machinery movement, noise and habitat loss, are also powerful confounding factors. Consequently, observed patterns cannot be conclusively attributed to ALAN alone, underscoring the need for further research in high‐value natural areas to clarify the specific effects of ALAN on nocturnal Lepidoptera.

## Author Contributions

**D. Trombin:** data curation (equal), formal analysis (equal), investigation (equal), methodology (equal), visualization (equal), writing – original draft (equal). **P. Mazzei:** validation (equal), writing – review and editing (equal). **I. Piccini:** methodology (equal), validation (equal), writing – review and editing (equal). **L. Dessì:** investigation (equal), visualization (equal), writing – review and editing (equal). **P. Grieco:** resources (equal), supervision (equal), writing – review and editing (equal). **E. Matechou:** formal analysis (equal), resources (equal), validation (equal), writing – review and editing (equal). **S. Bonelli:** funding acquisition (equal), methodology (equal), project administration (equal), resources (equal), supervision (equal), validation (equal), writing – review and editing (equal).

## Funding

This research was co‐funded by the Turin Euralpin Lyon Turin (TELT) company within the framework of the GREEN‐themed PhD pathways (Action IV.5) of the XXXVII Cycle PhD Programmes. Specifically, a dedicated pathway entitled “Development of protocols to assess the impact of major infrastructure projects on biodiversity” was established within the PhD Programme in Biological and Applied Biotechnological Sciences at the Department of Life Sciences and Systems Biology, University of Turin.

## Ethics Statement

The authors confirm that the ethical policies of the journal, as noted on the Journal's author guidelines page, have been adhered to.

## Conflicts of Interest

One author is employed by the Turin Euralpin Lyon Turin (TELT) company, which co‐founded the PhD project underlying this research. This author had no role in the study design, data collection, analysis, or interpretation. All other authors declare no competing interests.

## Supporting information


**Data S1:** ece374296‐sup‐0001‐DataS1.zip.

## Data Availability

All the required data are uploaded as supporting information (Data S1).

## References

[ece374296-bib-0001] Abrams, P. A. , O. Leimar , S. Nylin , and C. Wiklund . 1996. “The Effect of Flexible Growth Rates on Optimal Sizes and Development Times in a Seasonal Environment.” American Naturalist 147, no. 3: 381–395. 10.1086/285857.

[ece374296-bib-0002] Akaike, H. 1973. “Information Theory and an Extension of the Maximum Likelihood Principle.” In Second International Symposium on Information Theory, 267–281. Springer. 10.1007/978-1-4612-1694-0_15.

[ece374296-bib-0003] Angilletta, M. J. , R. S. Wilson , C. A. Navas , and R. S. James . 2003. “Tradeoffs and the Evolution of Thermal Reaction Norms.” Trends in Ecology & Evolution 18, no. 5: 234–240. 10.1016/S0169-5347(03)00087-9.

[ece374296-bib-0004] Basset, Y. , N. D. Springate , H. P. Aberlenc , and G. Delvare . 1996. “A Review of Methods for Sampling Arthropods in Tree Canopies.” In Canopy Arthropods, 35. Chapman and Hall.

[ece374296-bib-0005] Bates, A. J. , J. P. Sadler , D. Grundy , et al. 2014. “Garden and Landscape‐Scale Correlates of Moths of Differing Conservation Status: Significant Effects of Urbanization and Habitat Diversity.” PLoS One 9, no. 1: e86925. 10.1371/journal.pone.0086925. PMC3903603 24475197

[ece374296-bib-0006] Benedetti‐Cecchi, L. 2001. “Beyond BACI: Optimization of Environmental Sampling Designs Through Monitoring and Simulation.” Ecological Applications 11, no. 3: 783–799. 10.1890/1051-0761(2001)011[0783:BBOOES]2.0.CO;2.

[ece374296-bib-0007] Benítez‐López, A. , R. Alkemade , and P. A. Verweij . 2010. “The Impacts of Roads and Other Infrastructure on Mammal and Bird Populations: A Meta‐Analysis.” Biological Conservation 143, no. 6: 1307–1316. 10.1016/j.biocon.2010.02.009.

[ece374296-bib-0008] Bennie, J. , T. W. Davies , D. Cruse , R. Inger , and K. J. Gaston . 2018. “Artificial Light at Night Causes Top‐Down and Bottom‐Up Trophic Effects on Invertebrate Populations.” Journal of Applied Ecology 55, no. 6: 2698–2706. 10.1111/1365-2664.13240.

[ece374296-bib-0009] Bennie, J. , T. W. Davies , J. P. Duffy , R. Inger , and K. J. Gaston . 2014. “Contrasting Trends in Light Pollution Across Europe Based on Satellite Observed Night Time Lights.” Scientific Reports 4, no. 1: 3789. 10.1038/srep03789. PMC3896907 24445659

[ece374296-bib-0010] Berger, D. , and K. Gotthard . 2008. “Time Stress, Predation Risk and Diurnal–Nocturnal Foraging Trade‐Offs in Larval Prey.” Behavioral Ecology and Sociobiology 62, no. 10: 1655–1663. 10.1007/s00265-008-0594-4.

[ece374296-bib-0011] Berger, J. L. , M. Staab , M. Hartlieb , et al. 2024. “The Day After Mowing: Time and Type of Mowing Influence Grassland Arthropods.” Ecological Applications 34, no. 6: e3022. 10.1002/eap.3022. 39099295

[ece374296-bib-0012] Boggs, C. L. , and K. D. Freeman . 2005. “Larval Food Limitation in Butterflies: Effects on Adult Resource Allocation and Fitness.” Oecologia 144, no. 3: 353–361. 10.1007/s00442-005-0076-6. 15891831

[ece374296-bib-0013] Boyes, D. H. , D. M. Evans , R. Fox , M. S. Parsons , and M. J. O. Pocock . 2021a. “Is Light Pollution Driving Moth Population Declines? A Review of Causal Mechanisms Across the Life Cycle.” Insect Conservation and Diversity 14, no. 2: 167–187. 10.1111/icad.12447.

[ece374296-bib-0014] Boyes, D. H. , D. M. Evans , R. Fox , M. S. Parsons , and M. J. O. Pocock . 2021b. “Street Lighting Has Detrimental Impacts on Local Insect Populations.” Science Advances 7, no. 35: eabi8322. 10.1126/sciadv.abi8322. PMC8386932 34433571

[ece374296-bib-0015] Brock, A. 2023. “A Green Extractivist Railway? Exploring the Political Ecology of Europe's Largest Infrastructure Project.” Journal of Political Ecology 30, no. 1: 488–515. 10.2458/jpe.5637.

[ece374296-bib-0091] Brooks, M. E. , K. Kristensen , K. J. Van Benthem , et al. 2017. “glmmTMB Balances Speed and Flexibility Among Packages for Zero‐Inflated Generalized Linear Mixed Modeling.” R Journal 9, no. 2: 378–400.

[ece374296-bib-0016] Cheriyan, D. , and J. Choi . 2020. “A Review of Research on Particulate Matter Pollution in the Construction Industry.” Journal of Cleaner Production 254: 120077. 10.1016/j.jclepro.2020.120077.

[ece374296-bib-0017] Christie, A. P. , T. Amano , P. A. Martin , G. E. Shackelford , B. I. Simmons , and W. J. Sutherland . 2019. “Simple Study Designs in Ecology Produce Inaccurate Estimates of Biodiversity Responses.” Journal of Applied Ecology 56, no. 12: 2742–2754. 10.1111/1365-2664.13499.

[ece374296-bib-0018] Cinzano, P. , F. Falchi , and C. D. Elvidge . 2001. “The First World Atlas of the Artificial Night Sky Brightness.” Monthly Notices of the Royal Astronomical Society 328, no. 3: 689–707. 10.1046/j.1365-8711.2001.04882.x.

[ece374296-bib-0019] Clark, C. W. 1994. “Antipredator Behavior and the Asset‐Protection Principle.” Behavioral Ecology 5, no. 2: 159–170. 10.1093/beheco/5.2.159.

[ece374296-bib-0020] Coruh, S. , and H. Ozbek . 2002. “Biology, Host Plants and Damage of *Malacosoma neustria* (L.) (Lepidoptera: Lasiocampidae) in Erzurum Province of Turckey. Vol. 33, No. 3, 283–287. CABI Databases.” ISSN (Electronic): 2651‐5016.

[ece374296-bib-0021] Davies, T. W. , J. Bennie , and K. J. Gaston . 2012. “Street Lighting Changes the Composition of Invertebrate Communities.” Biology Letters 8, no. 5: 764–767. 10.1098/rsbl.2012.0216. PMC3440964 22628095

[ece374296-bib-0022] De Palma, A. , K. Sanchez‐Ortiz , P. A. Martin , et al. 2018. “Challenges With Inferring How Land‐Use Affects Terrestrial Biodiversity: Study Design, Time, Space and Synthesis.” In Advances in Ecological Research, vol. 58, 163–199. Elsevier. 10.1016/bs.aecr.2017.12.004.

[ece374296-bib-0023] Degen, T. , O. Mitesser , E. K. Perkin , et al. 2016. “Street Lighting: Sex‐Independent Impacts on Moth Movement.” Journal of Animal Ecology 85, no. 5: 1352–1360. 10.1111/1365-2656.12540. 27146262

[ece374296-bib-0024] Dominoni, D. , M. Quetting , and J. Partecke . 2013. “Artificial Light at Night Advances Avian Reproductive Physiology.” Proceedings of the Royal Society B: Biological Sciences 280, no. 1756: 20123017. 10.1098/rspb.2012.3017. PMC3574380 23407836

[ece374296-bib-0025] Falchi, F. , R. Furgoni , T. A. Gallaway , et al. 2019. “Light Pollution in USA and Europe: The Good, the Bad and the Ugly.” Journal of Environmental Management 248: 109227. 10.1016/j.jenvman.2019.06.128. 31362173

[ece374296-bib-0026] Flyvbjerg, B. 2014. “What You Should Know About Megaprojects and Why: An Overview.” Project Management Journal 45, no. 2: 6–19. 10.1002/pmj.21409.

[ece374296-bib-0027] Fox, R. 2013. “The Decline of Moths in Great Britain: A Review of Possible Causes.” Insect Conservation and Diversity 6, no. 1: 5–19. 10.1111/j.1752-4598.2012.00186.x.

[ece374296-bib-0028] Franzén, M. , and M. Johannesson . 2007. “Predicting Extinction Risk of Butterflies and Moths (Macrolepidoptera) From Distribution Patterns and Species Characteristics.” Journal of Insect Conservation 11, no. 4: 367–390. 10.1007/s10841-006-9053-6.

[ece374296-bib-0030] Gaston, K. J. , J. P. Duffy , S. Gaston , J. Bennie , and T. W. Davies . 2014. “Human Alteration of Natural Light Cycles: Causes and Ecological Consequences.” Oecologia 176, no. 4: 917–931. 10.1007/s00442-014-3088-2. PMC4226844 25239105

[ece374296-bib-0031] Gellert, P. K. , and B. D. Lynch . 2003. “Mega‐Projects as Displacements*.” International Social Science Journal 55, no. 175: 15–25. 10.1111/1468-2451.5501002.

[ece374296-bib-0032] Gili, F. , C. Fassone , A. Rolando , and S. Bertolino . 2024. “In the Spotlight: Bat Activity Shifts in Response to Intense Lighting of a Large Railway Construction Site.” Sustainability 16, no. 6: 2337. 10.3390/su16062337.

[ece374296-bib-0033] Gilliam, J. F. , and D. F. Fraser . 1987. “Habitat Selection Under Predation Hazard: Test of a Model With Foraging Minnows.” Ecology 68, no. 6: 1856–1862. 10.2307/1939877. 29357169

[ece374296-bib-0034] Grenis, K. , and S. M. Murphy . 2019. “Direct and Indirect Effects of Light Pollution on the Performance of an Herbivorous Insect.” Insect Science 26, no. 4: 770–776. 10.1111/1744-7917.12574. 29425403

[ece374296-bib-0035] Grenis, K. , B. Tjossem , and S. M. Murphy . 2015. “Predation of Larval Lepidoptera in Habitat Fragments Varies Spatially and Temporally but Is Not Affected by Light Pollution.” Journal of Insect Conservation 19, no. 3: 559–566. 10.1007/s10841-015-9777-2.

[ece374296-bib-0036] Groenendijk, D. , and W. N. Ellis . 2011. “The State of the Dutch Larger Moth Fauna.” Journal of Insect Conservation 15, no. 1–2: 95–101. 10.1007/s10841-010-9326-y.

[ece374296-bib-0037] Guetté, A. , L. Godet , M. Juigner , and M. Robin . 2018. “Worldwide Increase in Artificial Light at Night Around Protected Areas and Within Biodiversity Hotspots.” Biological Conservation 223: 97–103. 10.1016/j.biocon.2018.04.018.

[ece374296-bib-0038] Güneralp, B. , X. Xu , and W. Lin . 2021. “Infrastructure Development With(Out) Ecological Conservation: The Northern Forests in İstanbul.” Regional Environmental Change 21, no. 3: 86. 10.1007/s10113-021-01807-w.

[ece374296-bib-0039] Harrell, F. E. 2015. Regression Modeling Strategies: With Applications to Linear Models, Logistic and Ordinal Regression, and Survival Analysis. Springer International Publishing. 10.1007/978-3-319-19425-7.

[ece374296-bib-0092] Hartig, F. 2016. “DHARMa: Residual Diagnostics for Hierarchical (Multi‐Level/Mixed) Regression Models.” CRAN: Contributed Packages.

[ece374296-bib-0040] Hellmann, F. , and E. Bertaccini . 2004. I Macrolepidotteri della Valla di Susa. Italia Nord‐Occidentale (Alpi Cozie‐Graie). Museo Regionale di Scienze Naturali.

[ece374296-bib-0042] Hölker, F. , T. Moss , B. Griefahn , et al. 2010. “The Dark Side of Light: A Transdisciplinary Research Agenda for Light Pollution Policy.” Ecology and Society 15, no. 4: art13. 10.5751/ES-03685-150413.

[ece374296-bib-0043] Holyoak, M. , V. Jarosik , and I. Novák . 1997. “Weather‐Induced Changes in Moth Activity Bias Measurement of Long‐Term Population Dynamics From Light Trap Samples.” Entomologia Experimentalis et Applicata 83, no. 3: 329–335. 10.1046/j.1570-7458.1997.00188.x.

[ece374296-bib-0044] Hou, L. , Y. Xu , J. Dong , H.‐Y. Chong , R. Ren , and Z. Chen . 2024. “Reshaping Port‐City Relationships Through Underground Logistics System: A Mixed Qualitative Approach.” Cities 154: 105395. 10.1016/j.cities.2024.105395.

[ece374296-bib-0046] Kirichenko, N. I. , V. M. Petko , E. Magnoux , and C. Lopez‐Vaamonde . 2017. “Diversity and Distribution of Leaf Mining Insects on Birches (Betula Spp.) in Siberia.” Entomological Review 97, no. 2: 183–198. 10.1134/S0013873817020051.

[ece374296-bib-0047] Kristensen, C. O. 1994. “Investigations on the Natural Mortality of Eggs and Larvae of the Large White *Pieris brassicae* (L.) (Lep., Pieridae).” Journal of Applied Entomology 117, no. 1–5: 92–98. 10.1111/j.1439-0418.1994.tb00712.x.

[ece374296-bib-0048] Kronfeld‐Schor, N. , and T. Dayan . 2003. “Partitioning of Time as an Ecological Resource.” Annual Review of Ecology, Evolution, and Systematics 34, no. 1: 153–181. 10.1146/annurev.ecolsys.34.011802.132435.

[ece374296-bib-0049] Kumar, A. , and R. Shaw . 2020. “Transforming Rural Light and Dark Under Planetary Urbanisation: Comparing Ordinary Countrysides in India and the uk .” Transactions of the Institute of British Geographers 45, no. 1: 155–167. 10.1111/tran.12342.

[ece374296-bib-0050] Laurance, W. F. , A. Peletier‐Jellema , B. Geenen , et al. 2015. “Reducing the Global Environmental Impacts of Rapid Infrastructure Expansion.” Current Biology 25, no. 7: R259–R262. 10.1016/j.cub.2015.02.050. 25754645

[ece374296-bib-0051] Loewy, K. J. , A. L. Flansburg , K. Grenis , et al. 2013. “Life History Traits and Rearing Techniques for Fall Webworms ( *Hyphantria cunea* Drury) in Colorado.” Journal of the Lepidopterists' Society 67, no. 3: 196–205. 10.18473/lepi.v67i3.a6.

[ece374296-bib-0052] Lüdecke, D. , M. Ben‐Shachar , I. Patil , P. Waggoner , and D. Makowski . 2021. “Performance: An R Package for Assessment, Comparison and Testing of Statistical Models.” Journal of Open Source Software 6, no. 60: 3139. 10.21105/joss.03139.

[ece374296-bib-0053] Macgregor, C. J. , D. M. Evans , R. Fox , and M. J. O. Pocock . 2017. “The Dark Side of Street Lighting: Impacts on Moths and Evidence for the Disruption of Nocturnal Pollen Transport.” Global Change Biology 23, no. 2: 697–707. 10.1111/gcb.13371. 27251575

[ece374296-bib-0054] Maes, D. , and H. V. Dyck . 2001. “Butterfly Diversity Loss in Flanders (North Belgium): Europe's Worst Case Scenario?” Biological Conservation 99, no. 3: 263–276. 10.1016/S0006-3207(00)00182-8.

[ece374296-bib-0056] Merckx, T. , L. Marini , R. E. Feber , and D. W. Macdonald . 2012. “Hedgerow Trees and Extended‐Width Field Margins Enhance Macro‐Moth Diversity: Implications for Management.” Journal of Applied Ecology 49, no. 6: 1396–1404. 10.1111/j.1365-2664.2012.02211.x.

[ece374296-bib-0057] Merckx, T. , and H. Van Dyck . 2019. “Urbanization‐Driven Homogenization Is More Pronounced and Happens at Wider Spatial Scales in Nocturnal and Mobile Flying Insects.” Global Ecology and Biogeography 28, no. 10: 1440–1455. 10.1111/geb.12969.

[ece374296-bib-0058] Meyer, L. A. , and S. M. P. Sullivan . 2013. “Bright Lights, Big City: Influences of Ecological Light Pollution on Reciprocal Stream–Riparian Invertebrate Fluxes.” Ecological Applications 23, no. 6: 1322–1330. 10.1890/12-2007.1. 24147405

[ece374296-bib-0059] Nagy Pintérné, E. , and Z. Pödör . 2017. “The Effect of Artificial Lights on Nocturnal Macrolepidoptera (Lepidoptera: Macroheterocera) Communities.” Acta Silvatica et Lignaria Hungarica 13, no. 1: 41–54. 10.1515/aslh-2017-0003.

[ece374296-bib-0060] Neff, F. , Y. Chittaro , F. Korner‐Nievergelt , G. Litsios , E. Rey , and E. Knop . 2024. “Bimodal Seasonal Activity of Moths and Elevation, Weather and Land Use as Drivers of Their Diversity. Biodiversity.” 10.32942/X2NS6Q.

[ece374296-bib-0061] Nel, J. 2007. “Patrice Leraut.‐Papillons de nuit d'Europe. Volume 1, Bombyx, Lasiocampes, Sphinx, Ecailles…NAP Editions (Verrières‐le‐Buisson, France), Novembre 2006.” Bulletin de la Société Entomologique de France 112, no. 1: 72.

[ece374296-bib-0062] Ojija, F. , L. P. Lutambi , M. E. Mng'ong'o , G. Y. S. Mtui , and Z. S. Katambara . 2025. “Biodiversity Conservation and Construction Industry: Impacts, Regulatory Frameworks, and Challenges.” CABI Reviews 20: 0032. 10.1079/cabireviews.2025.0032.

[ece374296-bib-0063] Owens, A. C. S. , P. Cochard , J. Durrant , B. Farnworth , E. K. Perkin , and B. Seymoure . 2020. “Light Pollution Is a Driver of Insect Declines.” Biological Conservation 241: 108259. 10.1016/j.biocon.2019.108259.

[ece374296-bib-0064] Perkin, E. K. , F. Hölker , J. S. Richardson , J. P. Sadler , C. Wolter , and K. Tockner . 2011. “The Influence of Artificial Light on Stream and Riparian Ecosystems: Questions, Challenges, and Perspectives.” Ecosphere 2, no. 11: art122. 10.1890/ES11-00241.1.

[ece374296-bib-0065] Péter, Á. , G. Seress , K. Sándor , E. Vincze , K. P. Klucsik , and A. Liker . 2020. “The Effect of Artificial Light at Night on the Biomass of Caterpillars Feeding in Urban Tree Canopies.” Urban Ecosystems 23, no. 6: 1311–1319. 10.1007/s11252-020-00999-z.

[ece374296-bib-0066] Piccini, I. , M. Depetris , F. Paradiso , et al. 2023. “Macro‐Moth (Lepidoptera) Diversity of a Newly Shaped Ecological Corridor and the Surrounding Forest Area in the Western Italian Alps.” Diversity 15, no. 1: 95. 10.3390/d15010095.

[ece374296-bib-0067] Reavey, D. 1993. Why Body Size Matters to Caterpillars. Chapman & Hall Ltd ISBN (Hardback): 0‐412‐02681‐3.

[ece374296-bib-0068] Robineau, R. 2011. Guide des papillons nocturnes de France. Plus de 1620 espèces décreites et illustrées. 2ème édition ed, 287. Delachaux et Niestlé.

[ece374296-bib-0069] Sandil, S. , and R. Kumar . 2022. “Soil Contamination From Construction Projects.” In Ecological and Health Effects of Building Materials, edited by J. A. Malik and S. Marathe , 205–244. Springer International Publishing. 10.1007/978-3-030-76073-1_12.

[ece374296-bib-0070] Sibly, R. , P. Calow , and N. Nichols . 1985. “Are Patterns of Growth Adaptive?” Journal of Theoretical Biology 112, no. 3: 553–574. 10.1016/S0022-5193(85)80022-9. 3982053

[ece374296-bib-0072] Slansky, F., Jr. 1993. Nutritional Ecology: The Fundamental Quest for Nutrients. Chapman & Hall Ltd ISBN (Hardback): 978‐0‐412‐02681‐2.

[ece374296-bib-0073] Sloan, S. , M. J. Campbell , M. Alamgir , A. M. Lechner , J. Engert , and W. F. Laurance . 2019. “Trans‐National Conservation and Infrastructure Development in the Heart of Borneo.” PLoS One 14, no. 9: e0221947. 10.1371/journal.pone.0221947. PMC6750574 31532810

[ece374296-bib-0074] Stone, E. L. , G. Jones , and S. Harris . 2009. “Street Lighting Disturbs Commuting Bats.” Current Biology 19, no. 13: 1123–1127. 10.1016/j.cub.2009.05.058. 19540116

[ece374296-bib-0075] Thomas, J. A. 2005. “Monitoring Change in the Abundance and Distribution of Insects Using Butterflies and Other Indicator Groups.” Philosophical Transactions of the Royal Society, B: Biological Sciences 360, no. 1454: 339–357. 10.1098/rstb.2004.1585. PMC1569450 15814349

[ece374296-bib-0076] Thounaojam, N. , and B. Laishram . 2022. “Issues in Promoting Sustainability in Mega Infrastructure Projects: A Systematic Review.” Journal of Environmental Planning and Management 65, no. 8: 1349–1372. 10.1080/09640568.2021.1941810.

[ece374296-bib-0077] Trombin, D. , L. Dessì , and S. Bonelli . 2026. “The Impact of Artificial Light at Night on Moths (Ledpidoptera, Heterocera): A Review of the Literature and Future Research Directions.” Conservation Science and Practice 8: e703327. 10.1111/csp2.70327.

[ece374296-bib-0078] Twidle, A. M. , D. Barker , L. I. Pilkington , B. Fedrizzi , and D. M. Suckling . 2022. “Identification of Herbivore‐Induced Plant Volatiles From Selected Rubus Species Fed Upon by Raspberry Bud Moth (Heterocrossa Rubophaga) Larvae.” Phytochemistry 202: 113325. 10.1016/j.phytochem.2022.113325. 35843359

[ece374296-bib-0079] Uhl, B. , M. Wölfling , and C. Bässler . 2022. “Mediterranean Moth Diversity Is Sensitive to Increasing Temperatures and Drought Under Climate Change.” Scientific Reports 12, no. 1: 14473. 10.1038/s41598-022-18770-z. PMC9411567 36008549

[ece374296-bib-0093] Underwood, A. J. 1992. “Beyond BACI: The Detection of Environmental Impacts on Populations in the Real, but Variable, World.” Journal of Experimental Marine Biology and Ecology 161, no. 2: 145–178.

[ece374296-bib-0080] Underwood, A. J. 1994. “On Beyond BACI: Sampling Designs That Might Reliably Detect Environmental Disturbances.” Ecological Applications 4, no. 1: 3–15. 10.2307/1942110.

[ece374296-bib-0081] Van Geffen, K. G. , E. Van Eck , R. A. De Boer , et al. 2015. “Artificial Light at Night Inhibits Mating in a Geometrid Moth.” Insect Conservation and Diversity 8, no. 3: 282–287. 10.1111/icad.12116.

[ece374296-bib-0082] Van Grunsven, R. H. A. , J. R. Van Deijk , M. Donners , et al. 2020. “Experimental Light at Night Has a Negative Long‐Term Impact on Macro‐Moth Populations.” Current Biology 30, no. 12: R694–R695. 10.1016/j.cub.2020.04.083. 32574627

[ece374296-bib-0083] Van Klink, R. , M. H. M. Menz , H. Baur , et al. 2019. “Larval and Phenological Traits Predict Insect Community Response to Mowing Regime Manipulations.” Ecological Applications 29, no. 4: e01900. 10.1002/eap.1900. 30980442

[ece374296-bib-0084] Van Rees, C. B. , D. D. Hernández‐Abrams , M. Shudtz , et al. 2023. “Reimagining Infrastructure for a Biodiverse Future.” Proceedings of the National Academy of Sciences of the United States of America 120, no. 46: e2214334120. 10.1073/pnas.2214334120. PMC10655554 37931104

[ece374296-bib-0085] Wickham, H. , W. Chang , and M. H. Wickham . “Package ‘ggplot2’. Create Elegant Data Visualisation Using the Grammar of Graphics. Version, 2 (1), 1–189” (2016).

[ece374296-bib-0086] Wickramasinghe, L. P. , S. Harris , G. Jones , and N. Vaughan Jennings . 2004. “Abundance and Species Richness of Nocturnal Insects on Organic and Conventional Farms: Effects of Agricultural Intensification on Bat Foraging.” Conservation Biology 18, no. 5: 1283–1292. 10.1111/j.1523-1739.2004.00152.x.

[ece374296-bib-0087] Wickramasinghe, L. P. , S. Harris , G. Jones , and N. Vaughan . 2003. “Bat Activity and Species Richness on Organic and Conventional Farms: Impact of Agricultural Intensification.” Journal of Applied Ecology 40, no. 6: 984–993. 10.1111/j.1365-2664.2003.00856.x.

[ece374296-bib-0088] Wu, H. , J. Chen , J. Xu , et al. 2019. “Effects of Dam Construction on Biodiversity: A Review.” Journal of Cleaner Production 221: 480–489. 10.1016/j.jclepro.2019.03.001.

[ece374296-bib-0089] Zalucki, M. P. , A. R. Clarke , and S. B. Malcolm . 2002. “Ecology and Behavior of First Instar Larval Lepidoptera.” Annual Review of Entomology 47, no. 1: 361–393. 10.1146/annurev.ento.47.091201.145220. 11729079

[ece374296-bib-0090] Zuur, A. F. , E. N. Ieno , N. J. Walker , A. A. Saveliev , and G. M. Smith . 2009. Mixed Effects Models and Extensions in Ecology With R. 574, 574. Springer.

