# Supplementary material for: Nocturnal Lepidoptera Larvae in Mega‐Project: Habitat and Environmental Factors Over Construction Effects
Source: Ecol Evol. 2026 Sep 29;16(10):e74296. doi: 10.1002/ece3.74296 (PMC13623453; doi:10.1002/ece3.74296)
Supplement: Supplementary file 1 [file ECE3-16-e74296-s001.zip › ece374296-sup-0017-TableS1.docx]

**Table S1 -** Habitat type, elevation range, and dominant vegetation composition for each construction site and its associated control areas.

| Area | Type | Habitat | Elevation range  (m a.s.l.) | Dominant plant species |
| --- | --- | --- | --- | --- |
| Area 1 | Construction site | Dry ecotone | 1271–1334 | *Betula pendula*, *Rosa canina*, *Berberis* spp. |
| Area 1A | Control | Dry ecotone | 1186–1281 | *Betula pendula*, *Rosa canina*, *Amelanchier ovalis* |
| Area 1B | Control | Dry ecotone | 1194–1277 | *Betula pendula*, *Rosa canina*, *Amelanchier ovalis* |
| Area 1C | Control | Dry ecotone | 1110–1276 | *Betula pendula*, *Rosa canina*, *Amelanchier ovalis* |
| Area 2 | Construction site | Perifluvial | 1087–1095 | *Fraxinus excelsior*, *Alnus incana*, *Betula pendula* |
| Area 2A | Control | Perifluvial | 1072–1099 | *Fraxinus excelsior*, *Alnus incana*, *Rosa canina* |
| Area 3 | Construction site | Ecotone | 638–746 | *Rosa canina*, *Betula pendula*, *Quercus* spp. |
| Area 3A | Control | Ecotone | 749–923 | *Quercus* spp., *Rosa canina*, *Rubus* spp. |
| Area 3B | Control | Ecotone | 734–1088 | *Rubus* spp., *Betula pendula*, *Quercus* spp. |
| Area 3C | Control | Ecotone | 725–753 | *Rosa canina*, *Quercus* spp., *Rubus* spp. |
