# Supplementary material for: Nocturnal Lepidoptera Larvae in Mega‐Project: Habitat and Environmental Factors Over Construction Effects
Source: Ecol Evol. 2026 Sep 29;16(10):e74296. doi: 10.1002/ece3.74296 (PMC13623453; doi:10.1002/ece3.74296)
Supplement: Supplementary file 1 [file ECE3-16-e74296-s001.zip › ece374296-sup-0018-TableS2.docx]

**Table S2 –** Statistical summary of light intensity values measured in each sampling area.

| Area | Max | Min | Mean | Median | SE |
| --- | --- | --- | --- | --- | --- |
| Area 1 | 35.20 | 0.00 | 2.04 | 0.02 | 0.59 |
| Area 1A | 0.00 | 0.00 | 0.00 | 0.00 | 0.00 |
| Area 1B | 0.00 | 0.00 | 0.00 | 0.00 | 0.00 |
| Area 1C | 0.00 | 0.00 | 0.00 | 0.00 | 0.00 |
| Area 2 | 13.00 | 0.25 | 3.30 | 2.31 | 0.45 |
| Area 2A | 0.00 | 0.00 | 0.00 | 0.00 | 0.00 |
| Area 3 | 20.60 | 0.00 | 5.33 | 2.83 | 0.44 |
| Area 3A | 0.12 | 0.00 | 0.05 | 0.04 | 0.00 |
| Area 3B | 0.13 | 0.00 | 0.02 | 0.01 | 0.00 |
| Area 3C | 0.07 | 0.00 | 0.01 | 0.00 | 0.00 |
