# Supplementary material for: Nocturnal Lepidoptera Larvae in Mega‐Project: Habitat and Environmental Factors Over Construction Effects
Source: Ecol Evol. 2026 Sep 29;16(10):e74296. doi: 10.1002/ece3.74296 (PMC13623453; doi:10.1002/ece3.74296)
Supplement: Supplementary file 1 [file ECE3-16-e74296-s001.zip › ece374296-sup-0019-TableS3.docx]

**Table S3** – List of the plant species in the study areas. The same number of individual plants per species was sampled in each impacted site and its corresponding control sites to ensure a balanced sampling design.

| **Construction sites** | **Plant species** | **Number of plants sampled** |
| --- | --- | --- |
| Area 1, 1A, 1B, 1C | *Betula* spp. | 8 |
|  | *Fraxinus excelsior* | 3 |
|  | *Amelanchier* spp*.* | 4 |
|  | *Berberis vulgaris* | 3 |
|  | *Crataegus monogyna* | 2 |
|  | *Rosa canina* | 8 |
| Area 2, 2A | *Betula* spp. | 2 |
|  | *Fraxinus excelsior* | 4 |
|  | *Populus* spp. | 2 |
|  | *Hippophae* spp. | 2 |
|  | *Alnus* spp. | 3 |
|  | *Salix* spp. | 2 |
|  | *Rosa canina* | 2 |
| Area 3, 3A, 3B, 3C | *Betula* spp. | 4 |
|  | *Fraxinus excelsior* | 3 |
|  | *Populus tremula* | 2 |
|  | *Quercus* spp. | 4 |
|  | *Crataegus monogyna* | 2 |
|  | *Rubus* spp. | 4 |
|  | *Rosa canina* | 4 |
