# Supplementary material for: Nocturnal Lepidoptera Larvae in Mega‐Project: Habitat and Environmental Factors Over Construction Effects
Source: Ecol Evol. 2026 Sep 29;16(10):e74296. doi: 10.1002/ece3.74296 (PMC13623453; doi:10.1002/ece3.74296)
Supplement: Supplementary file 1 [file ECE3-16-e74296-s001.zip › ece374296-sup-0020-TableS4.docx]

**Table S4** – List of covariates tested through the models and levels included in each one.

| Covariate | Abbreviation | Levels |
| --- | --- | --- |
| Sampling session | Repl | 1, 2, 3, 4 |
| Type area | Typ_area | Industrial, control |
| Habitat | Hab | Ecotone, dry ecotone, perifluvial |
| Weather | Weather | Clear, cloudy, rain, variable |
| Wind | Wind | Absent, mild, moderate, strong, very strong |
| Moon phase | Ph_moon | Crescent, new, waning |
| Moon percentage | Per_moon | Numerical (%) |
| Construction years | Cons_year | 0, 2, 10, 15 (factorial) |
| Plant species | Sp_veg | Twelve levels |
| Slope | Slope | Numeric |
| Artificial light value | Slux | Numeric |
| Instar | Instar | I (immature), M (mature) |
| Status plant | Status_pl | Green, defoliated, dry |
| Mow | Mow | No, new, old |
