# Supplementary material for: Nocturnal Lepidoptera Larvae in Mega‐Project: Habitat and Environmental Factors Over Construction Effects
Source: Ecol Evol. 2026 Sep 29;16(10):e74296. doi: 10.1002/ece3.74296 (PMC13623453; doi:10.1002/ece3.74296)
Supplement: Supplementary file 1 [file ECE3-16-e74296-s001.zip › ece374296-sup-0021-TableS5.docx]

**Table S5 –** List of covariates included in the forward selection for species richness.

| Covariate | Abbreviation | Levels |
| --- | --- | --- |
| Replicate | Repl | 1, 2, 3, 4 |
| Weather | Weather | Clear, cloudy, rain, variable |
| Moon phase | Ph_moon | Crescent, new, waning |
| Type plant | Type_pl | Shrub, tree |
| Status plant | Status_pl | Green, defoliated, dry |
| Artificial light value | Slux | Numeric |
| Mow | Mow | No, new, old |
