# Supplementary material for: Nocturnal Lepidoptera Larvae in Mega‐Project: Habitat and Environmental Factors Over Construction Effects
Source: Ecol Evol. 2026 Sep 29;16(10):e74296. doi: 10.1002/ece3.74296 (PMC13623453; doi:10.1002/ece3.74296)
Supplement: Supplementary file 1 [file ECE3-16-e74296-s001.zip › ece374296-sup-0023-TableS7.docx]

**Table S7 –** Frequency distribution of the number of individuals recorded across sampling points.

| Number of individuals | 0 | 1 | 2 | 4 | 5 | 8 | 15 |
| --- | --- | --- | --- | --- | --- | --- | --- |
| Frequencies | 796 | 211 | 2 | 1 | 1 | 1 | 1 |
