# Supplementary material for: Nocturnal Lepidoptera Larvae in Mega‐Project: Habitat and Environmental Factors Over Construction Effects
Source: Ecol Evol. 2026 Sep 29;16(10):e74296. doi: 10.1002/ece3.74296 (PMC13623453; doi:10.1002/ece3.74296)
Supplement: Supplementary file 1 [file ECE3-16-e74296-s001.zip › ece374296-sup-0024-TableS8.docx]

**Table S8 –** List of **s**tatistical models ran during the forward selection of covariates to analyse presence/absence of larvae.

mod1 <- glmmTMB(pa ~ (1|point), family = "binomial", data = data4)

mod2 <- glmmTMB(pa ~ area + (1|point), family = "binomial", data = data4)

mod3 <- glmmTMB(pa ~ area + mow + (1|point), family = "binomial", data = data4)

mod4 <- glmmTMB(pa ~ area + mow + sp_veg + (1|point), family = "binomial", data = data4)

mod5 <- glmmTMB(pa ~ area + mow + sp_veg + wind + (1|point), family = "binomial", data = data4)
