# Supplementary material for: Nocturnal Lepidoptera Larvae in Mega‐Project: Habitat and Environmental Factors Over Construction Effects
Source: Ecol Evol. 2026 Sep 29;16(10):e74296. doi: 10.1002/ece3.74296 (PMC13623453; doi:10.1002/ece3.74296)
Supplement: Supplementary file 1 [file ECE3-16-e74296-s001.zip › ece374296-sup-0025-TableS9.docx]

**Table S9** – List of the AIC values obtained during the forward selection of the variable in the presence/absence analysis.

| **Model** | **AIC** |
| --- | --- |
| Model 1 | 962.809 |
| Model 2 | 928.620 |
| Model 3 | 910.399 |
| Model 4 | 901.263 |
| Model 5 | 892.151 |
