# Supplementary material for: Nocturnal Lepidoptera Larvae in Mega‐Project: Habitat and Environmental Factors Over Construction Effects
Source: Ecol Evol. 2026 Sep 29;16(10):e74296. doi: 10.1002/ece3.74296 (PMC13623453; doi:10.1002/ece3.74296)
Supplement: Supplementary file 1 [file ECE3-16-e74296-s001.zip › ece374296-sup-0026-TableS10.docx]

**Table S10** – List of **s**tatistical models ran during the forward selection of covariates to analyse species richness.

mod1 <- glmmTMB(n_sp ~ (1|point), family = "nbinom1", ziformula = ~ 1, data = rich_point)

mod2 <- glmmTMB(n_sp ~ repl + (1|point), family = "nbinom1", ziformula = ~ 1, data = rich_point)

mod3 <- glmmTMB(n_sp ~ area + (1|point), family = "nbinom1", ziformula = ~ 1, data = rich_point)

mod4 <- glmmTMB(n_sp ~ weather + (1|point), family = "nbinom1", ziformula = ~ 1, data = rich_point)

mod5 <- glmmTMB(n_sp ~ ph_moon + (1|point), family = "nbinom1", ziformula = ~ 1, data = rich_point)

mod6 <- glmmTMB(n_sp ~ Slux + (1|point), family = "nbinom1", ziformula = ~ 1, data = rich_point)

mod7 <- glmmTMB(n_sp ~ type_pl + (1|point), family = "nbinom1", ziformula = ~ 1, data = rich_point)

mod8 <- glmmTMB(n_sp ~ status_pl + (1|point), family = "nbinom1", ziformula = ~ 1, data = rich_point)

mod9 <- glmmTMB(n_sp ~ mow + (1|point), family = "nbinom1", ziformula = ~ 1, data = rich_point)

mod10 <- glmmTMB(n_sp ~ area + repl + (1|point), family = "nbinom1", ziformula = ~ 1, data = rich_point)

mod11 <- glmmTMB(n_sp ~ area + weather + (1|point), family = "nbinom1", ziformula = ~ 1, data = rich_point)

mod12 <- glmmTMB(n_sp ~ area + ph_moon + (1|point), family = "nbinom1", ziformula = ~ 1, data = rich_point)

mod13 <- glmmTMB(n_sp ~ area + Slux + (1|point), family = "nbinom1", ziformula = ~ 1, data = rich_point)

mod14 <- glmmTMB(n_sp ~ area + type_pl + (1|point), family = "nbinom1", ziformula = ~ 1, data = rich_point)

mod15 <- glmmTMB(n_sp ~ area + status_pl + (1|point), family = "nbinom1", ziformula = ~ 1, data = rich_point)

mod16 <- glmmTMB(n_sp ~ area + mow + (1|point), family = "nbinom1", ziformula = ~ 1, data = rich_point)

mod17 <- glmmTMB(n_sp ~ area + mow + repl + (1|point), family = "nbinom1", ziformula = ~ 1, data = rich_point)

mod18 <- glmmTMB(n_sp ~ area + mow + weather + (1|point), family = "nbinom1", ziformula = ~ 1, data = rich_point)

mod19 <- glmmTMB(n_sp ~ area + mow + ph_moon + (1|point), family = "nbinom1", ziformula = ~ 1, data = rich_point)

mod20 <- glmmTMB(n_sp ~ area + mow + Slux + (1|point), family = "nbinom1", ziformula = ~ 1, data = rich_point)

mod21 <- glmmTMB(n_sp ~ area + mow + type_pl + (1|point), family = "nbinom1", ziformula = ~ 1, data = rich_point)

mod22 <- glmmTMB(n_sp ~ area + mow + status_pl + (1|point), family = "nbinom1", ziformula = ~ 1, data = rich_point)

mod23 <- glmmTMB(n_sp ~ area + mow + status_pl + repl + (1|point), family = "nbinom1", ziformula = ~ 1, data = rich_point)

mod24 <- glmmTMB(n_sp ~ area + mow + status_pl + weather + (1|point), family = "nbinom1", ziformula = ~ 1, data = rich_point)

mod25 <- glmmTMB(n_sp ~ area + mow + status_pl + ph_moon + (1|point), family = "nbinom1", ziformula = ~ 1, data = rich_point)

mod26 <- glmmTMB(n_sp ~ area + mow + status_pl + Slux + (1|point), family = "nbinom1", ziformula = ~ 1, data = rich_point)

mod27 <- glmmTMB(n_sp ~ area + mow + status_pl + type_pl + (1|point), family = "nbinom1", ziformula = ~ 1, data = rich_point)

mod28 <- glmmTMB(n_sp ~ area + mow + status_pl + repl + weather + (1|point), family = "nbinom1", ziformula = ~ 1, data = rich_point)

mod29 <- glmmTMB(n_sp ~ area + mow + status_pl + repl + ph_moon + (1|point), family = "nbinom1", ziformula = ~ 1, data = rich_point)

mod30 <- glmmTMB(n_sp ~ area + mow + status_pl + repl + Slux + (1|point), family = "nbinom1", ziformula = ~ 1, data = rich_point)

mod31 <- glmmTMB(n_sp ~ area + mow + status_pl + repl + type_pl + (1|point), family = "nbinom1", ziformula = ~ 1, data = rich_point)

mod32 <- glmmTMB(n_sp ~ area + mow + status_pl + repl + (1|point), family = "nbinom1", ziformula = ~ 1, data = rich_point)
