# Supplementary material for: Nocturnal Lepidoptera Larvae in Mega‐Project: Habitat and Environmental Factors Over Construction Effects
Source: Ecol Evol. 2026 Sep 29;16(10):e74296. doi: 10.1002/ece3.74296 (PMC13623453; doi:10.1002/ece3.74296)
Supplement: Supplementary file 1 [file ECE3-16-e74296-s001.zip › ece374296-sup-0027-TableS11.docx]

**Table S11** – List of the AIC values obtained during the forward selection of the variable in the species richness analysis.

| **Modello** | **AIC** |
| --- | --- |
| Modello 1 | 1030.526 |
| Modello 2 | 1022.192 |
| Modello 3 | 999.661 |
| Modello 4 | 1033.506 |
| Modello 5 | 1026.012 |
| Modello 6 | 1029.255 |
| Modello 7 | 1031.379 |
| Modello 8 | 1024.239 |
| Modello 9 | 1021.269 |
| Modello 10 | 990.609 |
| Modello 11 | 1004.847 |
| Modello 12 | 1001.243 |
| Modello 13 | 999.927 |
| Modello 14 | 1000.688 |
| Modello 15 | 991.824 |
| Modello 16 | 989.955 |
| Modello 17 | 989.042 |
| Modello 18 | 995.453 |
| Modello 19 | 993.129 |
| Modello 20 | 990.243 |
| Modello 21 | 991.005 |
| Modello 22 | 985.883 |
| Modello 23 | 985.377 |
| Modello 24 | 991.231 |
| Modello 25 | 989.229 |
| Modello 26 | 986.061 |
| Modello 27 | 986.782 |
| Modello 28 | 990.557 |
| Modello 29 | 987.232 |
| Modello 30 | 985.510 |
| Modello 31 | 986.359 |
| Modello 32 | 985.377 |
