# Supplementary material for: Nocturnal Lepidoptera Larvae in Mega‐Project: Habitat and Environmental Factors Over Construction Effects
Source: Ecol Evol. 2026 Sep 29;16(10):e74296. doi: 10.1002/ece3.74296 (PMC13623453; doi:10.1002/ece3.74296)
Supplement: Supplementary file 1 [file ECE3-16-e74296-s001.zip › ece374296-sup-0028-TableS12.docx]

**Table S12 –** Results of univariate generalised linear mixed models testing the effect of ALAN (lux) on larval occurrence run prior to forward selection process.

| Response | Predictor | Esitmate | SE | z | p-vallue | IC 95% |
| --- | --- | --- | --- | --- | --- | --- |
| Larval occurrence | Lux | -0.0830 | 0.050 | -1.653 | 0.098 | [-0.182,0.015] |
