# Supplementary material for: Nocturnal Lepidoptera Larvae in Mega‐Project: Habitat and Environmental Factors Over Construction Effects
Source: Ecol Evol. 2026 Sep 29;16(10):e74296. doi: 10.1002/ece3.74296 (PMC13623453; doi:10.1002/ece3.74296)
Supplement: Supplementary file 1 [file ECE3-16-e74296-s001.zip › ece374296-sup-0030-TableS14.docx]

**Table S14 –** Results of univariate generalised linear mixed model testing the effect of ALAN (lux) on species richness run prior to forward selection process.

| Response | Predictor | Estimate | SE | z | p-value | IC 95% |
| --- | --- | --- | --- | --- | --- | --- |
| Species richness (n_sp) | ALAN (Slux) | -0.232 | 0.144 | -1.608 | 0.108 | [-0.515, 0.051] |
