# Supplementary material for: Nocturnal Lepidoptera Larvae in Mega‐Project: Habitat and Environmental Factors Over Construction Effects
Source: Ecol Evol. 2026 Sep 29;16(10):e74296. doi: 10.1002/ece3.74296 (PMC13623453; doi:10.1002/ece3.74296)
Supplement: Supplementary file 1 [file ECE3-16-e74296-s001.zip › ece374296-sup-0031-Supinfo_captions.docx]

**Figure S1 –** Puits d’Avrieux (Area 1) construction site in French study area.

**Figure S2 –** Villarodin-Bourget-Modane (Area 2) construction site in French study area.

**Figure S3 –** La Maddalena (Area 3) construction site in Italian study area.

**Figure S4 –** French study areas. In detail: Puits d’Avrieux (Area 1) and the three control areas (Area 1A, 1B, 1C); Villarodin-Bourget-Modane (Area 2) and related control area (Area 2A).

**Figure S5 -** Italian study areas. In details: La Maddalena construction site (Area 3) and linked control areas (Area 3A, 3B, 3C).

**Figure S6** – Sampling points per area (Area 3, 3A, 3B, 3C).

**Figure S7** – Sampling points per area (Area 1, 1A, 2B, 3C).

**Figure S8** – Sampling points per area (Area 2, 2A).

**Figure S9** – Number of individuals sampled per each area. The red labels indicate the construction sites.

**Figure S10 –** Predicted probability of moth larvae presence in different mowing conditions where “no” indicates there was no mowing activity in the sampling period, “new” indicates recent mowing activity, and “old” indicates past mowing activity.

**Figure S11 –** Predicted probability of moth larvae occurrence on different plant species.

**Figure S12 –** Predicted probabilities of moth larval presence across different slope exposures.

**Figure S13** – Species richness per sampling area; construction site labels are in red character.

**Figure S14** – Predicted species richness per plant status.

**Figure S15** – Predicted species richness per each sampling session.

**Figure S16** – Distribution of biomass on the logarithmic scale on different plant species.

**Table S1** – Habitat type, elevation range, and dominant vegetation composition for each construction site and its associated control areas.

**Table S2** – Statistical summary of light intensity values measured in each sampling area.

**Table S3 –** List of the plant species in the study areas. The same number of individual plants per species was sampled in each impacted site and its corresponding control sites to ensure a balanced sampling design.

**Table S4 –** List of covariates tested through the models and levels included in each one.

**Table S5 –** List of covariates included in the forward selection for species richness.

**Table S6 –** List of covariates included in the forward selection for biomass.

**Table S7** – Frequency distribution of the number of individuals recorded across sampling points.

**Table S8** – List of **s**tatistical models ran during the forward selection of covariates to analyse presence/absence of larvae.

**Table S9** – List of the AIC values obtained during the forward selection of the variable in the presence/absence analysis.

**Table S10 –** List of **s**tatistical models ran during the forward selection of covariates to analyse species richness.

**Table S11 –** List of the AIC values obtained during the forward selection of the variable in the species richness analysis.

**Table S12 –** Results of univariate generalised linear mixed models testing the effect of ALAN (lux) on larval occurrence run prior to forward selection process.

**Table S13** – Species list per each sampling area.

**Table S14** – Results of univariate generalised linear mixed model testing the effect of ALAN (lux) on species richness run prior to forward selection process.
